# Overcoming Immunological Challenges to Helper-Dependent Adenoviral Vector-Mediated Long-Term *CFTR* Expression in Mouse Airways

**DOI:** 10.3390/genes11050565

**Published:** 2020-05-18

**Authors:** Huibi Cao, Rongqi Duan, Jim Hu

**Affiliations:** 1 Program of Translational Medicine, The Hospital for Sick Children, Research Institute, 686 Bay Street, Toronto, ON M5G 0A4, Canada; huibi.cao@sickkids.ca (H.C.); cathleen.duan@sickkids.ca (R.D.); 2 Departments of Paediatrics and Laboratory Medicine and Pathobiology, University of Toronto, 1 King’s College Circle, Toronto, ON M5S 1A8, Canada

**Keywords:** cystic fibrosis, gene therapy, cyclophosphamide, transient immunosuppression

## Abstract

Cystic Fibrosis (CF) is caused by mutations in the cystic fibrosis transmembrane conductance regulator (*CFTR*) gene, and CF patients require life-long treatment. Although *CFTR* modulators show a great potential for treating most CF patients, some individuals may not tolerate the treatment. In addition, there is no effective therapy for patients with some rare *CFTR* mutations, such as class I CF mutations, which lead to a lack of *CFTR* protein production. Therefore, other therapeutic strategies, such as gene therapy, have to be investigated. Currently, immune responses to gene therapy vectors and transgene products are a major obstacle to applying CF gene therapy to clinical applications. In this study, we examined the effects of cyclophosphamide on the modulation of host immune responses and for the improvement of the *CFTR* transgene expression in the repeated delivery of helper-dependent adenoviral (HD-Ad) vectors to mouse lungs. We have found that cyclophosphamide significantly decreased the expression of T cell genes, such as CD3 (cluster of differentiation 3) and CD4, and reduced their infiltration into mouse lung tissues. We have also found that the levels of the anti-adenoviral antibody and neutralizing activity as well as B-cell infiltration into the mouse lung tissues were significantly reduced with this treatment. Correspondingly, the expression of the human *CFTR* transgene has been significantly improved with cyclophosphamide administration compared to the group with no treatment. These data suggest that the sustained expression of the human *CFTR* transgene in mouse lungs through repeated vector delivery can be achieved by transient immunosuppression.

## 1. Introduction

Cystic fibrosis (CF) is a life-long inherited disease that affects >70,000 patients globally; it is caused by underlying genetic mutations in the cystic fibrosis transmembrane conductance regulator (*CFTR*) gene [1]. New *CFTR*-modulator drugs show promise for up to 90% of patients, including patients with *CFTR* mutations for which early modulators are ineffective [2,3,4,5]. Although *CFTR* modulators show enormous potential for treating most CF patients, they are expensive and patients require lifetime treatments [6]. In addition, some patients may not tolerate the treatments. It is understandable that pharmaceutical approaches may not always effectively fix every malfunctioning human body caused by the same genetic defect [7]. Currently, there is no remedy for some patients with rare *CFTR* mutations, such as class I mutations, which lead to a lack of production of the *CFTR* protein [8]. Therefore, alternative therapeutic strategies, such as gene therapy, should be explored.

To date, more than 2600 gene therapy clinical trials have either been completed, are going to be, or have been approved worldwide [9]. Gene therapies for inherited immune disorders, hemophilia, eye and neurodegenerative disorders, and lymphoid cancers, have recently progressed to a stage where drugs have been approved in the United States and Europe [10]. Efficient gene delivery systems are essential to gene therapy to treat human genetic diseases. Genes can be delivered to target organs and cells with viral and non-viral vectors. As therapeutics, adenoviral (Ad) vectors represent one of the promising candidates among current available advance-therapy medical products [11]. Ad vector is still one of the most commonly utilized gene transfer vectors in a variety of potential applications for lung diseases (including inherited disease and cancer gene therapy). Ad-based vector can efficiently transduce dividing and non-dividing cells. They can be easily produced and purified in high titers.

The helper-dependent adenoviral (HD-Ad) vector has been developed based on adenovirus by deleting all viral genes. This makes the HD-Ad vector less immunogenic and allows it to have a large DNA-carrying capacity [12,13,14]. This unique feature of large capacity makes it ideal for the delivery of large genes, such as *CFTR*, together with gene regulating components. With more than 36 kb DNA payload capacity, HD-Ad vectors can easily carry gene editing components, such as guide RNA, CRISPR-Cas9, and therapeutic donor genes with gene-regulatory elements in the same vector [15,16,17]. Our previous studies have demonstrated that HD-Ad vectors are efficient for transducing airway surface epithelial cells and submucosal glands [18,19]. We have shown that abundant therapeutic *CFTR* protein expression at the apical membrane of airway epithelial cells as well as the submucosal glands of the conduct airways can be achieved by HD-Ad vector delivery to the lungs of mice and pigs. More importantly, HD-Ad vectors have been shown to transduce pig airway basal cells, which are considered as stem/progenitor cells [20,21].

However, the transduction of self-renewal tissues, such as airway epithelium with episomal vectors, requires repeated administration to achieve long-term gene correction [22]. Even considering stem/progenitor cell targeting with gene editing, repeated delivery may be still needed due to the current low in vivo gene-targeting efficiency. HD-Ad vectors, as other viral vectors, evoke host innate and adaptive immune responses against capsid proteins. Studies in experimental animals and clinical trials have shown that antibody and T cell responses can limit transgene expression duration and hinder the repeated administration of gene transfer [23]. Several approaches, including vector modification and host immune system modulations, have been investigated for minimizing immune responses [24,25,26]. All of these approaches have shown effects on the partial reduction in inflammation and immune reactions, but they are not fruitful in improving the duration of transgene expression. The aim of this study is to investigate how pharmacological agents can modulate the host immune system to allow the sustained expression of the *CFTR* gene from HD-Ad vectors in repeated delivery to mouse airways.

## 2. Materials and Methods

### 2.1. HD-Ad Vector Preparation and Delivery to Mice Lungs

HD-Ad-*CFTR* (helper-dependent adenoviral vector expressing the human *CFTR* gene) was prepared as described previously [27]. The purity of the HD-Ad vector preparations was determined by real-time q-PCR (quantitative polymerase chain reaction). For vector delivery, adult female C57Bl/6 mice (Charles River Laboratories, Wilmington, MA, USA) were anesthetized by isoflurane inhalation, and 20 µL of 1.5 × 10^10^ of HD-Ad-CFTR vector in PBS (phosphate buffered saline) with 40 µg/mL of DEAE-dextran (diethylaminoethyl-dextran) and 0.1% L-α-lysophosphatidylcholine (LPC) (Sigma-Aldrich, Oakville, ON, Canada) were placed in small drops onto one nostril from which they were aspirated into the lungs. All the mice, including the experiment and control groups, were given three rounds of HD-Ad-*CFTR* vector with the same dose at day 0 (d0), day 60 (d60), and day 120 (d120). Only the experiment group was injected intraperitoneally (ip) with cyclophosphamide (50 mg/kg) at 6 h before and 4 and 8 days after the vector delivery. At 3 and 33 days following the final round of vector delivery, the mice were sacrificed and blood samples were collected by cardiac puncture. A bronchoalveolar lavage was performed three times, each with 0.9 mL of phosphate-buffered saline. The lungs were collected for RNA isolation and histological analyses. Another group of mice received a single dose of 1.5 × 10^10^ HD-Ad-*CFTR* vectors with the same formula and procedure, and were sacrificed 3 days following the vector delivery. The animal studies were conducted following the Animal Use Protocol (#48813) approved by the Animal Care Committee of The Hospital for Sick Children, Toronto, Canada.

### 2.2. RNA Isolation and Real-Time RT-qPCR

Total RNA from mouse lungs was isolated using an RNeasy kit (a simplified technology for total RNA isolation) (Qiagen, Mississauga, ON, Canada) according to the manufacturer’s instructions (Invitrogen, Carlsbad, CA, USA), followed by DNase digestion. For SYBR Green real-time RT-PCR, the total RNA (1 µg) was reverse-transcribed using random hexamers and SuperScript II reverse transcriptase following the manufacturer’s protocol. The resulting templates (10 ng cDNA) were used for real-time PCR (ABI Prism 7500, Applied Biosystems, Foster City, CA, USA). For relative quantification, the PCR signals were compared between groups after normalization using 18S (TaqMan, Ribosomal RNA Control Reagents, Applied Biosystems, Foster City, CA, USA) as an internal reference. The following are primer sequences for the PCR analysis of vector *CFTR* and T cell marker genes: K18CFTR-F: CCTGAGTCCTGTCCTTTCTC, K18CFTR-RCGCTGTCTGTATCCTTTCCTC; mCD3e-F: GGACGATGCCGAGAACATTGA, mCD3e-R: CCAGGTGCTTATCATGCTTCTG; mCD4-F: GCCCTCATATACACACACCTGT, mCD4-R: GCAGCAGCAGCAGCAGCAA; mCD8a-F: GCTCAGTCATCAGCAACTCG, mCD8a-R: GTGAGGGAGTTCGCAGCACT.

### 2.3. Anti-Ad Antibody Titer Assay

A pan-specific (IgA, IgE, IgGs, IgM) ELISA for mouse anti-human Ad5 antibodies was performed, as previously described [19,28]. A 96-well ELISA plate (Corning Costar, Acton, MA, USA) was coated with 5 × 10^9^ viral particles of human Ad5 per well overnight at 4 °C in a 100 mM bicarbonate buffer at pH 9.6. The plate was then washed with TBS (Tris-buffered saline) and blocked with 3% BSA (bovine serum albumin) in TBS. Mouse bronchoalveolar lavage fluid (BALF) and serum diluted in 1:200 or 1:200,000 TBS, respectively, were added to the wells for overnight incubation. After washing with TBS, the plate was incubated with anti-mouse-Ig-biotin (BD Pharmingen, San Diego, CA, USA) and diluted in 1:5000 TBS for 3 hr at room temperature. The plate was washed again and incubated with avidin-alkaline phosphatase (Sigma-Aldrich, Oakville, ON, Canada), diluted 1:50,000 in TBS for 2 hr at room temperature. The plate was subsequently washed and incubated with 1 mg/mL p-nitrophenyl phosphate (Sigma-Aldrich, Oakville, ON, Canada) in 100 mM diethanolamine buffer at pH 9.8, containing 0.5 mM MgCl_2_, for 10 min at room temperature. The reaction was stopped by the addition of 25 µL of 0.2 M EDTA (ethylenediaminetetraacetic acid) and the optical density was read at 405 nm.

### 2.4. Neutralizing Abs Assay

The ability of the mouse heat-inactivated serum to block the Ad5 infection of HeLa cells was measured, as described previously [28]. The diluted BALF and serum was incubated with HD-Ad-LacZ and then infected HeLa cells. After 3 days, the cells were fixed and stained with an X-gal solution. The highest dilution that resulted in a minimum 50% reduction in blue cells was recorded. When no reduction was observed, the lowest dilution was conservatively assigned.

### 2.5. CDs Marker Staining

Immunohistochemistry staining was performed by the Pathology Core at The Toronto Centre for Phenogenomics. Tissue sections were submitted to heat-induced epitope retrieval with citrate buffer (pH 6.0) or with Tris-EDTA buffer (pH 9.0) for 7 min, followed by the quenching of endogenous peroxidase with 0.3% hydrogen peroxide in methanol. Non-specific antibody binding was blocked with 2.5% normal horse or goat serum (Vector, Laboratories, Burlingame, CA, USA), followed by incubation for 1 h in rat anti-CD3 at a 1:150 dilution (ab11089, Abcam, Toronto, ON, Canada), rabbit anti-CD4 at a 1:500 dilution (ab183685, Abcam, Toronto, ON, Canada), rabbit anti-CD8 at a 1:1000 dilution (ab209775, Abcam, Toronto, ON Canada), or rat anti-B220 at a 1:200 dilution (14-0452-82, Invitrogen, Mississauga, ON Canada). After washes, the tissue sections were incubated for 30 min with an ImmPRESS HRP (horseradish Peroxidase) reagent anti-rabbit or anti-rat IgG polymer (Vector, Laboratories, Burlingame, CA, USA), followed by a DAB (3′,3′-Diaminobenzidine) reagent.

### 2.6. Statistical Analysis

Five mice were used for each group. All the data on *CFTR* mRNA, cytokine gene expression, and antibody assays were tested with Prism one-way ANOVA multiple comparisons. The data were presented as mean ± SD (standard deviation). A *p* < 0.05 was considered significant. For the *CFTR* mRNA expression, the fold change was calculated according to Livak and Schmittgen [29].

## 3. Results

### 3.1. Scheme of Experiment Design for HD-Ad-CFTR Vector Delivery and Immunosuppressant Administration

**Figure 1:**
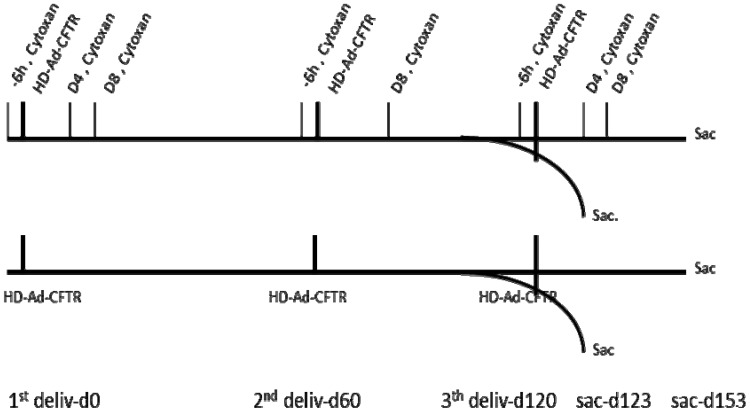
Time frame of the helper-dependent adenoviral (HD-Ad) vector transduction and cyclophosphamide administration. All the mice were nasally delivered HD-Ad-*CFTR* at a dose of 1.5 × 10^10^ viral particles in 3 rounds, each indicated by a heavy vertical line. The date of the first round of vector delivery is called d0. The second and third round was given at day 60 and day 120, respectively, following the first round. A cyclophosphamide (cytoxan) injection was administered at 6 h before (−6 h) and 4 (d4) and 8 days (d8) following the vector delivery. However, there was a glitch in the scheme where the cyclophosphamide injection at day 4 for the second round of vector delivery was missed. The samples were collected at days 3 and 33 after the last round of vector delivery, as shown in the scheme. Deliv: delivery; sac: sacrifice.

Our previous studies have shown that the HD-Ad-*CFTR* vector can efficiently transduce epithelial cells, but it induces significant inflammation and immune responses to the vector in mouse lungs [19]. In this study, we designed the experiment to investigate whether the expression of the transgene can be sustained through host immune system modulation in a model of repeated vector delivery. Three rounds of HD-Ad-*CFTR* vector were given intranasally to mice, with an interval of 2 months for each round. The immunosuppressant cyclophosphamide was administrated 6 h before vector delivery to block the initiation of inflammation and immune reaction. To maintain transiently the immunosuppression of the innate and subsequent adaptive immune responses, cyclophosphamide was given at day 4 and day 8 after the vector transduction. The immunosuppressant was administrated with a similar schedule for each round of vector delivery. As shown in Figure 1, the transgene expression and immune responses were analyzed at two time points, day 123 and day 153 following the first round of vector delivery.

### 3.2. Cyclophosphamide Reduced T Cell Gene Expression

**Figure 2:**
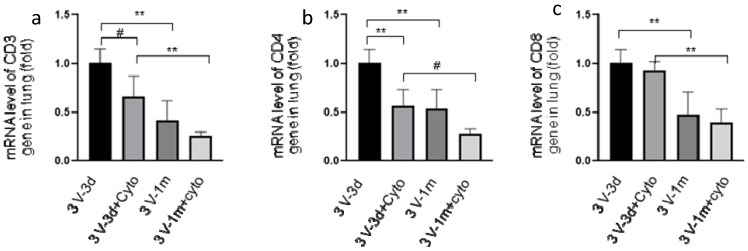
CD3 (cluster of differentiation 3) (**a**), CD4 (**b**), and CD8 (**c**) gene expression in mouse lungs determined by real-time RT-qPCR. RNA was isolated from mouse lung tissues, which were collected at days 3 and 33 after the last round of vector transduction from both groups of mice with or without cyclophosphamide treatments. The expression of CD3 (left panel), CD4 (middle panel), and CD8 (right panel) genes was normalized with 18S. 3V-3d: samples collected at day 3 following the third round of vector delivery; 3V-3d+cyto: samples collected from mice with the same time frame as those of 3v-3d but with cyclophosphamide treatments; 3V-1m: samples collected from mice in one month following the last round of vector delivery; 3v-1m+cyto: samples collected from mice with the same time frame as those of 3V-1m, but with cyclophosphamide treatments. Data are presented as mean ± SD (standard deviation), *n* = 5 for all groups. #: *p* < 0.05; **: *p* < 0.01.

**Figure 3:**
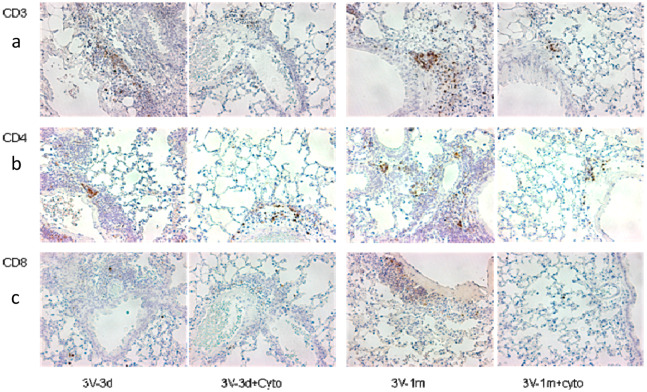
CD3 (**a**), CD4 (**b**), and CD8 (**c**) protein expression in mouse lungs. IHC (immunohistochemical) staining was performed in paraffin sections of mouse lungs and stained with antibodies against CD3, CD4, and CD8. The positive cells are shown in brown color. The sample labelling is the same as in Figure 2.

To investigate the effect of cyclophosphamide on T cells, CD3, CD4, and CD8 expression in mouse lungs was analyzed by real-time RT-qPCR. As shown in Figure 2, we observed that cyclophosphamide reduced the CD3 and CD4 gene expression by 34% and 44%, respectively, at day 3 following the last round of vector delivery compared to the mice without immunosuppression. At day 33, the CD3 and CD4 gene expression was further reduced by 38% and 47% in the cyclophosphamide-treated group. However, at this time point the difference in levels of expression between the groups with or without cyclophosphamide treatments was not statistically significant. This could be due to the small number of mice used in the experiment. Interestingly, the CD3, CD4, and CD8 gene expression decreased significantly between 3 and 33 days after the last round of vector delivery in both groups with or without cyclophosphamide administration (Figure 2). The CD3, CD4, and CD8 expression in the group with cyclophosphamide administration was reduced by 61%, 50%, and 57%, respectively, while their expression in the group without cyclophosphamide administration was reduced by 58%, 46%, and 53%, respectively. Nevertheless, these data suggested that transient immunosuppression can significantly block CD3 and CD4 gene expression. Our results were further confirmed by immunohistochemistry staining of the mouse lung tissues with antibodies against CD3, CD4, and CD8, as shown in Figure 3.

### 3.3. Cyclophosphamide Greatly Reduced B Cell Responses Induced by Repeated Vector Delivery

**Figure 4:**
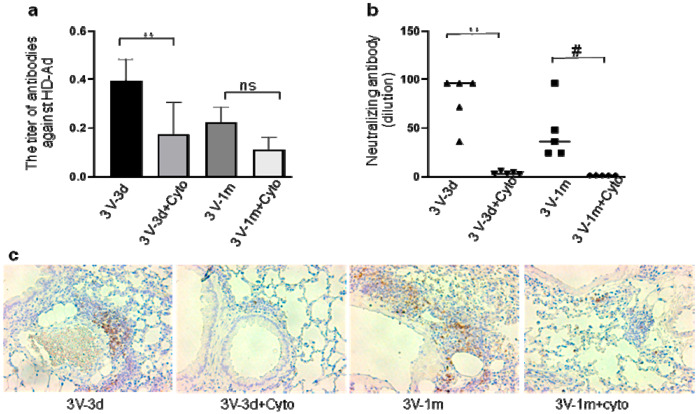
Host humoral immune responses to vector delivery. (**a**) The titer of anti-adenoviral antibodies in mouse bronchoalveolar lavage fluid (BALF). The total anti-Ad antibodies (IgA, IgE, IgGs, IgM) were detected with ELISA in all groups. Data were presented as mean ± SD (standard deviation). (**b**) Neutralizing antibody in mouse BALF. *n* = 5, #: *p* < 0.05; **: *p* < 0.01. (**c**) B-cell presence in mouse lungs. B-cells were detected in mouse lungs with antibodies against B220 by immunohistochemistry staining. The positive cells are shown in brown color. ns, no significant difference.

Our previous studies reported that cyclophosphamide can block humoral immune responses to vector delivery in mouse airways with reduced antibody production. In this study, repeated HD-Ad-*CFTR* vector delivery was expected to induce stronger immune responses to HD-Ad vector and transgene products. To test whether cyclophosphamide could still efficiently block B cell activation and functioning, we analyzed anti-adenoviral antibody and neutralizing antibodies at day 3 and day 33 after the last round of vector delivery in mouse BALF (Figure 4) and serum (Supplementary Figure S1). As shown in Figure 4a, the anti-Ad total antibodies in cyclophosphamide-treated mice were significantly lower compared to the group with no treatment at day 3. This means that cyclophosphamide administration efficiently prevented B cell function from the previous two rounds of HD-Ad-*CFTR* delivery. In contrast to CD3, CD4, and CD8 gene expression, the levels of anti-Ad antibodies were not decreased with time from day 3 to day 33 after the last round of vector delivery, when cyclophosphamide was not used.

In addition, at both days 3 and 33, the BALF- and serum-neutralizing antibody was greatly reduced by the cyclophosphamide administration (Figure 4b and Supplementary Figure S1b). This indicates that cyclophosphamide reduced the neutralizing antibody production from every round of vector transduction. Congruently, IHC staining with antibodies against B220 showed less infiltration of B220-positive cells in the lungs of mice treated with cyclophosphamide compared to the group without the treatment (Figure 4c). These data suggest that cyclophosphamide treatment during vector delivery can significantly block B cell activation induced by every round of vector transduction.

### 3.4. Transgene Expression was Improved Significantly by Immunosuppressant

**Figure 5:**
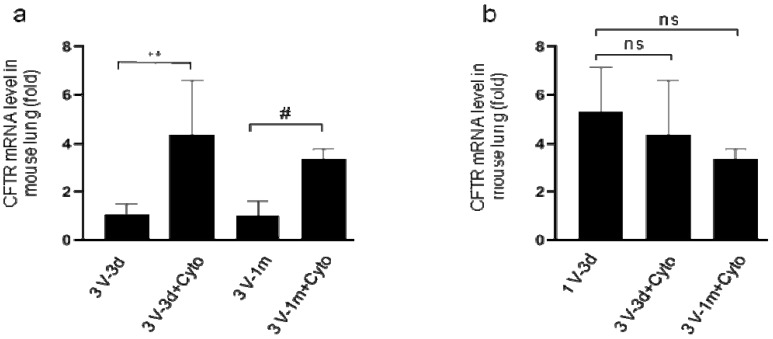
Expression of human cystic fibrosis transmembrane conductance regulator (*CFTR*) mRNA in mouse lungs assessed by real-time RT-qPCR. (**a**) *CFTR* mRNA levels in lung tissues of mice at days 3 and 33 following the last round of HD-Ad-*CFTR* delivery with or without cyclophosphamide treatments. The transgene expression was normalized with 18S. Data were presented as mean ± SD. *n* = 5; #: *p* < 0.05; **: *p* < 0.01. (**b**) *CFTR* mRNA levels in mouselung tissues from 3 days of single dose delivery (1V-3d), and at days 3 (3V-3d) and 33 (3V-33d) of 3 dose delivery following the last round of transduction with cyclophosphamide treatment. Data were presented as mean ± SD. Ns indicates no significant difference.

The goal of this study was to improve the long-term expression of transgene with cyclophosphamide treatment by blocking the airway inflammation and immune reaction to vector and transgene products. As shown in Figure 5a, the cyclophosphamide treatment improved the vector *CFTR* gene expression at both days 3 and 33 following the last round of vector delivery by 4.1 and 3.3 fold, respectively, compared to the vector-only group. The high level of human *CFTR* transgene expression at day 3 after the last round of transduction indicates that the cyclophosphamide treatment strongly reduced the vector-neutralizing activity in the previous rounds of vector delivery. We also observed that the *CFTR* expression level was not significantly reduced from day 3 to day 33 after the last round of delivery in the cyclophosphamide-treated group. This suggests that the cyclophosphamide treatment significantly preserved vector transduced cells. Finally, in this study of repeated vector delivery, the *CFTR* transgene expression level in cyclophosphamide-treated mice at days 123 and 153 from the first round of vector delivery remained at 83% and 63%, respectively, of that of the mice that received a single dose of vector delivery at day 3 (Figure 5b). These data clearly demonstrate that transient cyclophosphamide treatments help the targeting cells retain a relatively high level of sustained expression of the transgene in mouse lungs.

## 4. Discussion

In this repeated gene delivery study, we found the immunosuppressant cyclophosphamide significantly blocked the adaptive immune responses induced by the HD-Ad vector, showing a reduced T cell gene expression and T cell and B cell infiltration in mouse lungs. The transient immunosuppression resulted in a significant level of transgene expression at day 3 after three rounds of HD-Ad vector transduction and maintained a relatively high level of *CFTR* gene expression at 33 days after the last round of delivery. The slightly lower level of transgene expression in mice at day 33 may partially result from epithelial cells’ turn over. These data demonstrated that the sustained expression of the transgene in mouse lungs can be achieved by the repeated delivery of HD-Ad-*CFTR* vectors with a transient cyclophosphamide administration.

The sustained expression of transgene at therapeutic levels (achieving lifelong expression) in the terminal-differentiated cells of epithelia, which are capable of self-renewal, requires the repeated administration of vectors. Immune responses against vectors and transgene products are one of the major obstacles to sustained transgene expression in repeated vector delivery. Strategies to control unwanted immune responses to transgene products and vectors are needed to achieve the sustained expression of therapeutic genes in the lung. Lungs have their own set of immune cells which function as the frontline of immunity for the airway. Adaptive immune responses can be triggered by vector antigens or vector-encoded proteins during gene delivery. We and others have shown that gene transfer with Ad or HD-Ad vectors at single doses locally or systemically resulted in the activation of T cells and humoral immune responses to transgene products and vector antigens [30]. The repeated administration of most viral vectors is hindered by the toxicity of T cells and neutralizing or opsonizing the Abs induced by the vectors.

Thus, strategies including immunosuppression, induction of immune tolerance, or modification of viral capsids have been tested in vector delivery [22]. For example, Seregin et al. reported that transient pre-treatment with glucocorticoid can significantly reduce innate and adaptive immune responses to Ad vector with systemic delivery without a transgene expression reduction in mouse liver [31]. Immune suppressant prednisone has also been shown to reduce the extent of intramuscular T-cell infiltrates in AAV-treated muscles, which may aid in achieving long-term transgene expression through promoting PD1-mediated programmed T-cell death [32]. In addition, viral vector serotype switching [33] and capsid engineering [34] have been evaluated for viral vector readministration. The repeated liver gene transfer to adult mice with mutations in the liver-specific UGT1 gene by AAV serotype switching, upon neonatal administration, resulted in the lifelong correction of total bilirubin levels in the mice [35].

Cyclophosphamide is a potent immunosuppressive agent used clinically in autoimmune disorders and the transplantation of allogeneic bone marrow or solid organs to establish peripheral allograft tolerance and suppress autoreactive T cells [36]. Our previous study found that cyclophosphamide treatment significantly improved transgene expression at day 3 following HD-Ad readministration [19]. In the repeated airway transduction mouse model, the transient use of cyclophosphamide during HD-vector administration significantly reduced the expression of the CD3 and CD4 gene and the infiltration of these cells in the targeting organ. This indicated that cyclophosphamide may cause T cell death besides its anti-mitotic and anti-replicative effects [36]. Strauss et al. have reported that the effect of cyclophosphamide on T cells is its ability to induce apoptosis, which is a fundamental difference from other immunosuppressive agents.

Cyclophosphamide up-regulated Fas (CD95) expression, triggering activation-induced cell death. Comparing the effects of other drugs such as dexamethasone and rapamycin on human peripheral blood T cells, cytotoxic cell lines, and Jurkat cells, only cyclophosphamide triggered cell death [37,38]. The induction of apoptosis is also a key mechanism to eliminate activated T cells during the termination of an immune response.

In addition to T cell effects, cyclophosphamide also affects B cell proliferation and plasma cell antibody production [39]. In this study, HD-Ad vector transduction resulted in anti-Ad antibody production. Such antibodies can bind to and prevent viral vectors from entering target cells, thereby reducing the transgene expression. Transient cyclophosphamide administration significantly prevented anti-Ad antibody production and reduced the neutralizing activity. This reduction is expected to improve the vector transduction efficiency from the next round of vector delivery and sustained transgene expression. These studies established the important concept that the transient suppression of host immune responses is efficient for the sustained expression of transgene in mouse lungs in repeated HD-Ad vector delivery.

However, one limitation of this study is that we are not sure whether our results with mice can be translated into clinical applications, because there are differences between mice and humans in the immune system. These differences include: activation of immune responses to challenge, the balance of leukocyte subsets, Toll-like receptors, and antibody subsets [40,41]. Therefore, the safety, dosage, and scheduling of cyclophosphamide administration should be further evaluated in large animal models. Cyclophosphamide has immunosuppressive and immunomodulatory properties. Considering the risk of infection for persons with CF, the immunosuppressive function of cyclophosphamide and its effect on vector transduction efficiency should be tested in proper CF animal models. In this study, we did not investigate whether cyclophosphamide affects host innate immune responses. However, our previous work has shown that there was no significant change in cytokine production to HD-Ad vector with cyclophosphamide treatment [19]. Strategies with the combination of different immunosuppressive agents to target specifically innate and adaptive immune responses at different stages may also be important. In addition, the scheduling of administration is of particular importance for the immunological effect [36]. We observed that the expression of CD3, CD4, and CD 8 genes goes down significantly with time, one month after the last round of delivery with or without cyclophosphamide treatment. This observation suggests a window for scheduling the following round of vector administration in order to avoid the peak of adaptive immune responses.

## 5. Conclusions

In summary, the immunosuppressant cyclophosphamide can modulate the host immune system and allow for a more sustained expression of the *CFTR* gene in repeated vector delivery to mouse airways. In combination with a gene-editing system to target airway stem cells, sustained expression with a therapeutic level of transgene may be achievable in the future.

## Supplementary Materials

The following are available online at https://www.mdpi.com/2073-4425/11/5/565/s1, Figure S1: Anti-adenoviral antibodies in mouse serum. (a) The total anti-Ad antibodies (IgA, IgE, IgGs, IgM) were detected with ELISA in all groups. Data were presented as mean ± SD. (b) Neutralizing antibody in mouse sera. *n* = 5, #: *p* < 0.05; **: *p* < 0.01.
