# Supplementary material for: Overcoming Immunological Challenges to Helper-Dependent Adenoviral Vector-Mediated Long-Term CFTR Expression in Mouse Airways
Source: Genes (Basel). 2020 May 18;11(5):565. doi: 10.3390/genes11050565 (PMC7291004; doi:10.3390/genes11050565)
Supplement: Supplementary file 1 [file genes-11-00565-s001.pdf]

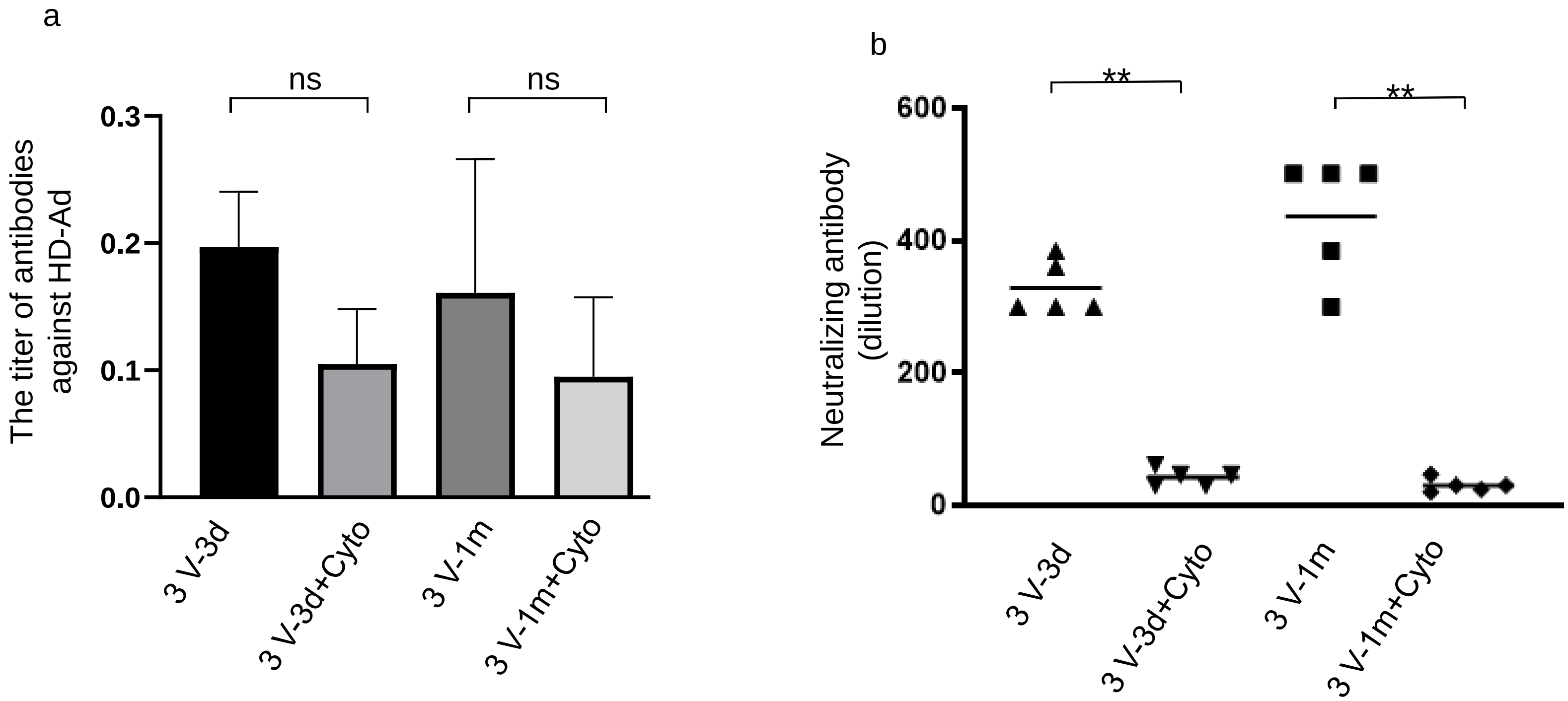

Supplementary figure 1. Anti-adenoviral antibodies in mouse serum. a) The total anti-Ad antibodies (IgA, IgE, IgGs, IgM) were detected with ELISA in all groups. Data were presented as mean ± SD. b) Neutralizing antibody in mouse sera. n=5, #: $p<0.05$; **: $p< 0.01$.
